# Supplementary material for: Pyruvate Dehydrogenase Kinase 1 inhibition mediated oxidative phosphorylation enhancement in cartilage promotes osteoarthritis progression
Source: BMC Musculoskelet Disord. 2023 Jul 20;24:597. doi: 10.1186/s12891-023-06585-6 (PMC10357736; doi:10.1186/s12891-023-06585-6)
Supplement: Supplementary file 1 — Additional file 1. [file 12891_2023_6585_MOESM1_ESM.zip › KEGG.Gsea.1653622432464/KEGG_MAPK_SIGNALING_PATHWAY.html]

Details for gene set KEGG\_MAPK\_SIGNALING\_PATHWAY[GSEA]

|  || Dataset | 55457\_collapsed\_to\_symbols.cls.cls #OA\_versus\_normal.cls.cls #OA\_versus\_normal\_repos |
| Phenotype | cls.cls#OA\_versus\_normal\_repos |
| Upregulated in class | normal |
| GeneSet | KEGG\_MAPK\_SIGNALING\_PATHWAY |
| Enrichment Score (ES) | -0.3075551 |
| Normalized Enrichment Score (NES) | -1.5049174 |
| Nominal p-value | 0.0013755158 |
| FDR q-value | 0.22092903 |
| FWER p-Value | 0.899 |
Table: GSEA Results Summary

  

Fig 1: Enrichment plot: KEGG\_MAPK\_SIGNALING\_PATHWAY      
 Profile of the Running ES Score & Positions of GeneSet Members on the Rank Ordered List

  

| SYMBOL | TITLE | RANK IN GENE LIST | RANK METRIC SCORE | RUNNING ES | CORE ENRICHMENT || 1 | FGF14 | fibroblast growth factor 14 [Source:HGNC Symbol;Acc:HGNC:3671] | 427 | 0.695 | -0.0248 | No |
| 2 | RRAS | RAS related [Source:HGNC Symbol;Acc:HGNC:10447] | 505 | 0.658 | -0.0225 | No |
| 3 | PLA2G12A | phospholipase A2 group XIIA [Source:HGNC Symbol;Acc:HGNC:18554] | 600 | 0.623 | -0.0219 | No |
| 4 | HSPA2 | heat shock protein family A (Hsp70) member 2 [Source:HGNC Symbol;Acc:HGNC:5235] | 662 | 0.603 | -0.0190 | No |
| 5 | MAPK9 | mitogen-activated protein kinase 9 [Source:HGNC Symbol;Acc:HGNC:6886] | 721 | 0.588 | -0.0160 | No |
| 6 | MAPK3 | mitogen-activated protein kinase 3 [Source:HGNC Symbol;Acc:HGNC:6877] | 757 | 0.579 | -0.0113 | No |
| 7 | PRKACB | protein kinase cAMP-activated catalytic subunit beta [Source:HGNC Symbol;Acc:HGNC:9381] | 771 | 0.576 | -0.0050 | No |
| 8 | TAB2 | TGF-beta activated kinase 1 (MAP3K7) binding protein 2 [Source:HGNC Symbol;Acc:HGNC:17075] | 877 | 0.549 | -0.0062 | No |
| 9 | FGF18 | fibroblast growth factor 18 [Source:HGNC Symbol;Acc:HGNC:3674] | 969 | 0.525 | -0.0067 | No |
| 10 | HSPA1L | heat shock protein family A (Hsp70) member 1 like [Source:HGNC Symbol;Acc:HGNC:5234] | 1139 | 0.488 | -0.0137 | No |
| 11 | AKT1 | AKT serine/threonine kinase 1 [Source:HGNC Symbol;Acc:HGNC:391] | 1192 | 0.478 | -0.0117 | No |
| 12 | CACNA2D3 | calcium voltage-gated channel auxiliary subunit alpha2delta 3 [Source:HGNC Symbol;Acc:HGNC:15460] | 1228 | 0.471 | -0.0084 | No |
| 13 | PTPRR | protein tyrosine phosphatase receptor type R [Source:HGNC Symbol;Acc:HGNC:9680] | 1352 | 0.450 | -0.0124 | No |
| 14 | STMN1 | stathmin 1 [Source:HGNC Symbol;Acc:HGNC:6510] | 1389 | 0.444 | -0.0095 | No |
| 15 | MAP2K5 | mitogen-activated protein kinase kinase 5 [Source:HGNC Symbol;Acc:HGNC:6845] | 1391 | 0.444 | -0.0039 | No |
| 16 | NTF3 | neurotrophin 3 [Source:HGNC Symbol;Acc:HGNC:8023] | 1398 | 0.443 | 0.0013 | No |
| 17 | CACNA2D2 | calcium voltage-gated channel auxiliary subunit alpha2delta 2 [Source:HGNC Symbol;Acc:HGNC:1400] | 1431 | 0.439 | 0.0044 | No |
| 18 | FGF23 | fibroblast growth factor 23 [Source:HGNC Symbol;Acc:HGNC:3680] | 1449 | 0.436 | 0.0087 | No |
| 19 | MAP3K7 | mitogen-activated protein kinase kinase kinase 7 [Source:HGNC Symbol;Acc:HGNC:6859] | 1701 | 0.399 | -0.0060 | No |
| 20 | FGF9 | fibroblast growth factor 9 [Source:HGNC Symbol;Acc:HGNC:3687] | 1833 | 0.380 | -0.0115 | No |
| 21 | PTPN7 | protein tyrosine phosphatase non-receptor type 7 [Source:HGNC Symbol;Acc:HGNC:9659] | 1864 | 0.377 | -0.0090 | No |
| 22 | ECSIT | ECSIT signaling integrator [Source:HGNC Symbol;Acc:HGNC:29548] | 1890 | 0.373 | -0.0062 | No |
| 23 | DUSP14 | dual specificity phosphatase 14 [Source:HGNC Symbol;Acc:HGNC:17007] | 1921 | 0.370 | -0.0038 | No |
| 24 | CD14 | CD14 molecule [Source:HGNC Symbol;Acc:HGNC:1628] | 1928 | 0.369 | 0.0004 | No |
| 25 | CRK | "CRK proto-oncogene, adaptor protein [Source:HGNC Symbol;Acc:HGNC:2362]" | 2018 | 0.360 | -0.0020 | No |
| 26 | TNF | tumor necrosis factor [Source:HGNC Symbol;Acc:HGNC:11892] | 2098 | 0.350 | -0.0037 | No |
| 27 | HSPB1 | heat shock protein family B (small) member 1 [Source:HGNC Symbol;Acc:HGNC:5246] | 2105 | 0.349 | 0.0003 | No |
| 28 | ARRB1 | arrestin beta 1 [Source:HGNC Symbol;Acc:HGNC:711] | 2116 | 0.348 | 0.0039 | No |
| 29 | GNG12 | G protein subunit gamma 12 [Source:HGNC Symbol;Acc:HGNC:19663] | 2168 | 0.342 | 0.0043 | No |
| 30 | MAP2K4 | mitogen-activated protein kinase kinase 4 [Source:HGNC Symbol;Acc:HGNC:6844] | 2214 | 0.337 | 0.0051 | No |
| 31 | CRKL | "CRK like proto-oncogene, adaptor protein [Source:HGNC Symbol;Acc:HGNC:2363]" | 2283 | 0.329 | 0.0039 | No |
| 32 | MAPK14 | mitogen-activated protein kinase 14 [Source:HGNC Symbol;Acc:HGNC:6876] | 2352 | 0.320 | 0.0026 | No |
| 33 | EGF | epidermal growth factor [Source:HGNC Symbol;Acc:HGNC:3229] | 2399 | 0.315 | 0.0031 | No |
| 34 | TGFB1 | transforming growth factor beta 1 [Source:HGNC Symbol;Acc:HGNC:11766] | 2490 | 0.303 | -0.0002 | No |
| 35 | RASA1 | RAS p21 protein activator 1 [Source:HGNC Symbol;Acc:HGNC:9871] | 2506 | 0.302 | 0.0025 | No |
| 36 | MAP4K2 | mitogen-activated protein kinase kinase kinase kinase 2 [Source:HGNC Symbol;Acc:HGNC:6864] | 2543 | 0.298 | 0.0035 | No |
| 37 | MAPK13 | mitogen-activated protein kinase 13 [Source:HGNC Symbol;Acc:HGNC:6875] | 2544 | 0.298 | 0.0073 | No |
| 38 | PLA2G4A | phospholipase A2 group IVA [Source:HGNC Symbol;Acc:HGNC:9035] | 2656 | 0.284 | 0.0022 | No |
| 39 | LAMTOR3 | "late endosomal/lysosomal adaptor, MAPK and MTOR activator 3 [Source:HGNC Symbol;Acc:HGNC:15606]" | 2674 | 0.283 | 0.0045 | No |
| 40 | CACNA1F | calcium voltage-gated channel subunit alpha1 F [Source:HGNC Symbol;Acc:HGNC:1393] | 2726 | 0.278 | 0.0040 | No |
| 41 | FGFR3 | fibroblast growth factor receptor 3 [Source:HGNC Symbol;Acc:HGNC:3690] | 2812 | 0.269 | 0.0008 | No |
| 42 | FGF20 | fibroblast growth factor 20 [Source:HGNC Symbol;Acc:HGNC:3677] | 2849 | 0.265 | 0.0013 | No |
| 43 | PDGFRA | platelet derived growth factor receptor alpha [Source:HGNC Symbol;Acc:HGNC:8803] | 2943 | 0.256 | -0.0027 | No |
| 44 | NTRK1 | neurotrophic receptor tyrosine kinase 1 [Source:HGNC Symbol;Acc:HGNC:8031] | 3015 | 0.248 | -0.0051 | No |
| 45 | CACNG4 | calcium voltage-gated channel auxiliary subunit gamma 4 [Source:HGNC Symbol;Acc:HGNC:1408] | 3065 | 0.244 | -0.0059 | No |
| 46 | CASP3 | caspase 3 [Source:HGNC Symbol;Acc:HGNC:1504] | 3202 | 0.230 | -0.0137 | No |
| 47 | PLA2G10 | phospholipase A2 group X [Source:HGNC Symbol;Acc:HGNC:9029] | 3315 | 0.220 | -0.0197 | No |
| 48 | MAP4K1 | mitogen-activated protein kinase kinase kinase kinase 1 [Source:HGNC Symbol;Acc:HGNC:6863] | 3328 | 0.219 | -0.0179 | No |
| 49 | ELK1 | ETS transcription factor ELK1 [Source:HGNC Symbol;Acc:HGNC:3321] | 3357 | 0.216 | -0.0173 | No |
| 50 | RPS6KA1 | ribosomal protein S6 kinase A1 [Source:HGNC Symbol;Acc:HGNC:10430] | 3389 | 0.213 | -0.0170 | No |
| 51 | MAPKAPK3 | MAPK activated protein kinase 3 [Source:HGNC Symbol;Acc:HGNC:6888] | 3483 | 0.204 | -0.0217 | No |
| 52 | CACNA1I | calcium voltage-gated channel subunit alpha1 I [Source:HGNC Symbol;Acc:HGNC:1396] | 3642 | 0.190 | -0.0318 | No |
| 53 | PLA2G3 | phospholipase A2 group III [Source:HGNC Symbol;Acc:HGNC:17934] | 3648 | 0.190 | -0.0297 | No |
| 54 | RPS6KA4 | ribosomal protein S6 kinase A4 [Source:HGNC Symbol;Acc:HGNC:10433] | 3702 | 0.185 | -0.0316 | No |
| 55 | PRKCA | protein kinase C alpha [Source:HGNC Symbol;Acc:HGNC:9393] | 3728 | 0.183 | -0.0312 | No |
| 56 | PPP3CA | protein phosphatase 3 catalytic subunit alpha [Source:HGNC Symbol;Acc:HGNC:9314] | 3803 | 0.176 | -0.0348 | No |
| 57 | MAX | MYC associated factor X [Source:HGNC Symbol;Acc:HGNC:6913] | 3804 | 0.176 | -0.0325 | No |
| 58 | CACNG2 | calcium voltage-gated channel auxiliary subunit gamma 2 [Source:HGNC Symbol;Acc:HGNC:1406] | 3819 | 0.174 | -0.0314 | No |
| 59 | FAS | Fas cell surface death receptor [Source:HGNC Symbol;Acc:HGNC:11920] | 3828 | 0.173 | -0.0298 | No |
| 60 | RAC3 | Rac family small GTPase 3 [Source:HGNC Symbol;Acc:HGNC:9803] | 3849 | 0.171 | -0.0292 | No |
| 61 | PAK1 | p21 (RAC1) activated kinase 1 [Source:HGNC Symbol;Acc:HGNC:8590] | 3861 | 0.169 | -0.0279 | No |
| 62 | FASLG | Fas ligand [Source:HGNC Symbol;Acc:HGNC:11936] | 3931 | 0.162 | -0.0312 | No |
| 63 | RELA | "RELA proto-oncogene, NF-kB subunit [Source:HGNC Symbol;Acc:HGNC:9955]" | 3942 | 0.161 | -0.0300 | No |
| 64 | MAPT | microtubule associated protein tau [Source:HGNC Symbol;Acc:HGNC:6893] | 3976 | 0.158 | -0.0305 | No |
| 65 | CACNA1S | calcium voltage-gated channel subunit alpha1 S [Source:HGNC Symbol;Acc:HGNC:1397] | 4031 | 0.153 | -0.0328 | No |
| 66 | GADD45A | growth arrest and DNA damage inducible alpha [Source:HGNC Symbol;Acc:HGNC:4095] | 4081 | 0.148 | -0.0348 | No |
| 67 | MEF2C | myocyte enhancer factor 2C [Source:HGNC Symbol;Acc:HGNC:6996] | 4218 | 0.135 | -0.0438 | No |
| 68 | MAPK7 | mitogen-activated protein kinase 7 [Source:HGNC Symbol;Acc:HGNC:6880] | 4396 | 0.121 | -0.0563 | No |
| 69 | CACNA1G | calcium voltage-gated channel subunit alpha1 G [Source:HGNC Symbol;Acc:HGNC:1394] | 4422 | 0.119 | -0.0567 | No |
| 70 | FGF1 | fibroblast growth factor 1 [Source:HGNC Symbol;Acc:HGNC:3665] | 4436 | 0.118 | -0.0562 | No |
| 71 | NFKB1 | nuclear factor kappa B subunit 1 [Source:HGNC Symbol;Acc:HGNC:7794] | 4626 | 0.102 | -0.0698 | No |
| 72 | TAOK3 | TAO kinase 3 [Source:HGNC Symbol;Acc:HGNC:18133] | 4633 | 0.101 | -0.0690 | No |
| 73 | CACNA2D1 | calcium voltage-gated channel auxiliary subunit alpha2delta 1 [Source:HGNC Symbol;Acc:HGNC:1399] | 4747 | 0.091 | -0.0768 | No |
| 74 | FGF8 | fibroblast growth factor 8 [Source:HGNC Symbol;Acc:HGNC:3686] | 4761 | 0.090 | -0.0767 | No |
| 75 | CACNG1 | calcium voltage-gated channel auxiliary subunit gamma 1 [Source:HGNC Symbol;Acc:HGNC:1405] | 4773 | 0.089 | -0.0764 | No |
| 76 | DUSP3 | dual specificity phosphatase 3 [Source:HGNC Symbol;Acc:HGNC:3069] | 4857 | 0.083 | -0.0819 | No |
| 77 | FGF5 | fibroblast growth factor 5 [Source:HGNC Symbol;Acc:HGNC:3683] | 4990 | 0.074 | -0.0914 | No |
| 78 | FGF16 | fibroblast growth factor 16 [Source:HGNC Symbol;Acc:HGNC:3672] | 5013 | 0.072 | -0.0922 | No |
| 79 | PLA2G2D | phospholipase A2 group IID [Source:HGNC Symbol;Acc:HGNC:9033] | 5056 | 0.069 | -0.0946 | No |
| 80 | FGF13 | fibroblast growth factor 13 [Source:HGNC Symbol;Acc:HGNC:3670] | 5070 | 0.067 | -0.0948 | No |
| 81 | TGFBR1 | transforming growth factor beta receptor 1 [Source:HGNC Symbol;Acc:HGNC:11772] | 5280 | 0.050 | -0.1107 | No |
| 82 | PPP3CB | protein phosphatase 3 catalytic subunit beta [Source:HGNC Symbol;Acc:HGNC:9315] | 5311 | 0.046 | -0.1124 | No |
| 83 | FGFR4 | fibroblast growth factor receptor 4 [Source:HGNC Symbol;Acc:HGNC:3691] | 5351 | 0.044 | -0.1150 | No |
| 84 | FGF7 | fibroblast growth factor 7 [Source:HGNC Symbol;Acc:HGNC:3685] | 5386 | 0.041 | -0.1171 | No |
| 85 | NTRK2 | neurotrophic receptor tyrosine kinase 2 [Source:HGNC Symbol;Acc:HGNC:8032] | 5431 | 0.038 | -0.1201 | No |
| 86 | TP53 | tumor protein p53 [Source:HGNC Symbol;Acc:HGNC:11998] | 5511 | 0.032 | -0.1259 | No |
| 87 | MECOM | MDS1 and EVI1 complex locus [Source:HGNC Symbol;Acc:HGNC:3498] | 5622 | 0.022 | -0.1343 | No |
| 88 | TAB1 | TGF-beta activated kinase 1 (MAP3K7) binding protein 1 [Source:HGNC Symbol;Acc:HGNC:18157] | 5684 | 0.017 | -0.1390 | No |
| 89 | GRB2 | growth factor receptor bound protein 2 [Source:HGNC Symbol;Acc:HGNC:4566] | 5697 | 0.016 | -0.1397 | No |
| 90 | MAP3K13 | mitogen-activated protein kinase kinase kinase 13 [Source:HGNC Symbol;Acc:HGNC:6852] | 5728 | 0.013 | -0.1419 | No |
| 91 | TRAF6 | TNF receptor associated factor 6 [Source:HGNC Symbol;Acc:HGNC:12036] | 5761 | 0.011 | -0.1443 | No |
| 92 | MKNK1 | MAPK interacting serine/threonine kinase 1 [Source:HGNC Symbol;Acc:HGNC:7110] | 5830 | 0.007 | -0.1496 | No |
| 93 | IKBKB | inhibitor of nuclear factor kappa B kinase subunit beta [Source:HGNC Symbol;Acc:HGNC:5960] | 5914 | 0.001 | -0.1561 | No |
| 94 | MAPKAPK5 | MAPK activated protein kinase 5 [Source:HGNC Symbol;Acc:HGNC:6889] | 6094 | -0.012 | -0.1701 | No |
| 95 | RAC2 | Rac family small GTPase 2 [Source:HGNC Symbol;Acc:HGNC:9802] | 6098 | -0.013 | -0.1702 | No |
| 96 | RAF1 | "Raf-1 proto-oncogene, serine/threonine kinase [Source:HGNC Symbol;Acc:HGNC:9829]" | 6187 | -0.019 | -0.1769 | No |
| 97 | TGFB3 | transforming growth factor beta 3 [Source:HGNC Symbol;Acc:HGNC:11769] | 6293 | -0.027 | -0.1848 | No |
| 98 | CACNA1C | calcium voltage-gated channel subunit alpha1 C [Source:HGNC Symbol;Acc:HGNC:1390] | 6367 | -0.033 | -0.1902 | No |
| 99 | FLNC | filamin C [Source:HGNC Symbol;Acc:HGNC:3756] | 6408 | -0.036 | -0.1929 | No |
| 100 | PRKCB | protein kinase C beta [Source:HGNC Symbol;Acc:HGNC:9395] | 6453 | -0.039 | -0.1959 | No |
| 101 | FLNB | filamin B [Source:HGNC Symbol;Acc:HGNC:3755] | 6499 | -0.042 | -0.1989 | No |
| 102 | RAP1A | "RAP1A, member of RAS oncogene family [Source:HGNC Symbol;Acc:HGNC:9855]" | 6580 | -0.048 | -0.2046 | No |
| 103 | CACNB3 | calcium voltage-gated channel auxiliary subunit beta 3 [Source:HGNC Symbol;Acc:HGNC:1403] | 6753 | -0.059 | -0.2174 | No |
| 104 | PLA2G5 | phospholipase A2 group V [Source:HGNC Symbol;Acc:HGNC:9038] | 6772 | -0.061 | -0.2181 | No |
| 105 | MAPK1 | mitogen-activated protein kinase 1 [Source:HGNC Symbol;Acc:HGNC:6871] | 6853 | -0.068 | -0.2235 | No |
| 106 | PDGFRB | platelet derived growth factor receptor beta [Source:HGNC Symbol;Acc:HGNC:8804] | 6914 | -0.073 | -0.2273 | No |
| 107 | DUSP7 | dual specificity phosphatase 7 [Source:HGNC Symbol;Acc:HGNC:3073] | 6966 | -0.077 | -0.2304 | No |
| 108 | MAP3K2 | mitogen-activated protein kinase kinase kinase 2 [Source:HGNC Symbol;Acc:HGNC:6854] | 6976 | -0.078 | -0.2301 | No |
| 109 | RAP1B | "RAP1B, member of RAS oncogene family [Source:HGNC Symbol;Acc:HGNC:9857]" | 6980 | -0.078 | -0.2293 | No |
| 110 | TGFB2 | transforming growth factor beta 2 [Source:HGNC Symbol;Acc:HGNC:11768] | 6985 | -0.079 | -0.2286 | No |
| 111 | PPP3CC | protein phosphatase 3 catalytic subunit gamma [Source:HGNC Symbol;Acc:HGNC:9316] | 7031 | -0.082 | -0.2311 | No |
| 112 | MAP3K1 | mitogen-activated protein kinase kinase kinase 1 [Source:HGNC Symbol;Acc:HGNC:6848] | 7066 | -0.085 | -0.2327 | No |
| 113 | RPS6KA3 | ribosomal protein S6 kinase A3 [Source:HGNC Symbol;Acc:HGNC:10432] | 7092 | -0.087 | -0.2336 | No |
| 114 | FGF2 | fibroblast growth factor 2 [Source:HGNC Symbol;Acc:HGNC:3676] | 7183 | -0.095 | -0.2395 | No |
| 115 | RAC1 | Rac family small GTPase 1 [Source:HGNC Symbol;Acc:HGNC:9801] | 7192 | -0.096 | -0.2389 | No |
| 116 | MAP2K6 | mitogen-activated protein kinase kinase 6 [Source:HGNC Symbol;Acc:HGNC:6846] | 7246 | -0.099 | -0.2418 | No |
| 117 | ARRB2 | arrestin beta 2 [Source:HGNC Symbol;Acc:HGNC:712] | 7255 | -0.100 | -0.2411 | No |
| 118 | GNA12 | G protein subunit alpha 12 [Source:HGNC Symbol;Acc:HGNC:4380] | 7294 | -0.102 | -0.2428 | No |
| 119 | NRAS | "NRAS proto-oncogene, GTPase [Source:HGNC Symbol;Acc:HGNC:7989]" | 7300 | -0.102 | -0.2419 | No |
| 120 | NLK | nemo like kinase [Source:HGNC Symbol;Acc:HGNC:29858] | 7336 | -0.105 | -0.2433 | No |
| 121 | FGFR2 | fibroblast growth factor receptor 2 [Source:HGNC Symbol;Acc:HGNC:3689] | 7374 | -0.108 | -0.2449 | No |
| 122 | PLA2G2E | phospholipase A2 group IIE [Source:HGNC Symbol;Acc:HGNC:13414] | 7486 | -0.117 | -0.2521 | No |
| 123 | CACNA1E | calcium voltage-gated channel subunit alpha1 E [Source:HGNC Symbol;Acc:HGNC:1392] | 7540 | -0.121 | -0.2548 | No |
| 124 | NGF | nerve growth factor [Source:HGNC Symbol;Acc:HGNC:7808] | 7572 | -0.125 | -0.2556 | No |
| 125 | CACNB1 | calcium voltage-gated channel auxiliary subunit beta 1 [Source:HGNC Symbol;Acc:HGNC:1401] | 7589 | -0.126 | -0.2552 | No |
| 126 | CACNA1H | calcium voltage-gated channel subunit alpha1 H [Source:HGNC Symbol;Acc:HGNC:1395] | 7635 | -0.129 | -0.2571 | No |
| 127 | FGF3 | fibroblast growth factor 3 [Source:HGNC Symbol;Acc:HGNC:3681] | 7637 | -0.130 | -0.2556 | No |
| 128 | BDNF | brain derived neurotrophic factor [Source:HGNC Symbol;Acc:HGNC:1033] | 7705 | -0.134 | -0.2591 | No |
| 129 | PAK2 | p21 (RAC1) activated kinase 2 [Source:HGNC Symbol;Acc:HGNC:8591] | 7762 | -0.139 | -0.2618 | No |
| 130 | CACNA1A | calcium voltage-gated channel subunit alpha1 A [Source:HGNC Symbol;Acc:HGNC:1388] | 7774 | -0.140 | -0.2609 | No |
| 131 | DUSP10 | dual specificity phosphatase 10 [Source:HGNC Symbol;Acc:HGNC:3065] | 7775 | -0.140 | -0.2591 | No |
| 132 | IL1A | interleukin 1 alpha [Source:HGNC Symbol;Acc:HGNC:5991] | 7842 | -0.144 | -0.2624 | No |
| 133 | HSPA1A | heat shock protein family A (Hsp70) member 1A [Source:HGNC Symbol;Acc:HGNC:5232] | 7876 | -0.146 | -0.2632 | No |
| 134 | FGFR1 | fibroblast growth factor receptor 1 [Source:HGNC Symbol;Acc:HGNC:3688] | 7905 | -0.148 | -0.2635 | No |
| 135 | HRAS | "HRas proto-oncogene, GTPase [Source:HGNC Symbol;Acc:HGNC:5173]" | 7977 | -0.153 | -0.2671 | No |
| 136 | AKT3 | AKT serine/threonine kinase 3 [Source:HGNC Symbol;Acc:HGNC:393] | 8008 | -0.156 | -0.2675 | No |
| 137 | SOS2 | SOS Ras/Rho guanine nucleotide exchange factor 2 [Source:HGNC Symbol;Acc:HGNC:11188] | 8058 | -0.160 | -0.2693 | No |
| 138 | RPS6KA6 | ribosomal protein S6 kinase A6 [Source:HGNC Symbol;Acc:HGNC:10435] | 8071 | -0.161 | -0.2682 | No |
| 139 | MAP3K14 | mitogen-activated protein kinase kinase kinase 14 [Source:HGNC Symbol;Acc:HGNC:6853] | 8100 | -0.163 | -0.2683 | No |
| 140 | PPM1A | "protein phosphatase, Mg2+/Mn2+ dependent 1A [Source:HGNC Symbol;Acc:HGNC:9275]" | 8125 | -0.165 | -0.2681 | No |
| 141 | MAPK12 | mitogen-activated protein kinase 12 [Source:HGNC Symbol;Acc:HGNC:6874] | 8161 | -0.168 | -0.2687 | No |
| 142 | BRAF | "B-Raf proto-oncogene, serine/threonine kinase [Source:HGNC Symbol;Acc:HGNC:1097]" | 8171 | -0.169 | -0.2672 | No |
| 143 | TNFRSF1A | TNF receptor superfamily member 1A [Source:HGNC Symbol;Acc:HGNC:11916] | 8196 | -0.171 | -0.2669 | No |
| 144 | PLA2G1B | phospholipase A2 group IB [Source:HGNC Symbol;Acc:HGNC:9030] | 8232 | -0.173 | -0.2675 | No |
| 145 | TGFBR2 | transforming growth factor beta receptor 2 [Source:HGNC Symbol;Acc:HGNC:11773] | 8241 | -0.174 | -0.2659 | No |
| 146 | TRAF2 | TNF receptor associated factor 2 [Source:HGNC Symbol;Acc:HGNC:12032] | 8322 | -0.180 | -0.2699 | No |
| 147 | RASGRP1 | RAS guanyl releasing protein 1 [Source:HGNC Symbol;Acc:HGNC:9878] | 8352 | -0.182 | -0.2699 | No |
| 148 | RPS6KA2 | ribosomal protein S6 kinase A2 [Source:HGNC Symbol;Acc:HGNC:10431] | 8397 | -0.185 | -0.2710 | No |
| 149 | CHP2 | calcineurin like EF-hand protein 2 [Source:HGNC Symbol;Acc:HGNC:24927] | 8573 | -0.199 | -0.2822 | No |
| 150 | PRKCG | protein kinase C gamma [Source:HGNC Symbol;Acc:HGNC:9402] | 8582 | -0.200 | -0.2803 | No |
| 151 | ATF2 | activating transcription factor 2 [Source:HGNC Symbol;Acc:HGNC:784] | 8592 | -0.201 | -0.2784 | No |
| 152 | MAP3K4 | mitogen-activated protein kinase kinase kinase 4 [Source:HGNC Symbol;Acc:HGNC:6856] | 8637 | -0.203 | -0.2793 | No |
| 153 | GADD45G | growth arrest and DNA damage inducible gamma [Source:HGNC Symbol;Acc:HGNC:4097] | 8671 | -0.206 | -0.2793 | No |
| 154 | CACNG3 | calcium voltage-gated channel auxiliary subunit gamma 3 [Source:HGNC Symbol;Acc:HGNC:1407] | 8677 | -0.207 | -0.2770 | No |
| 155 | DAXX | death domain associated protein [Source:HGNC Symbol;Acc:HGNC:2681] | 8926 | -0.226 | -0.2937 | No |
| 156 | MAPK8 | mitogen-activated protein kinase 8 [Source:HGNC Symbol;Acc:HGNC:6881] | 8956 | -0.228 | -0.2930 | No |
| 157 | RASGRF1 | Ras protein specific guanine nucleotide releasing factor 1 [Source:HGNC Symbol;Acc:HGNC:9875] | 8982 | -0.230 | -0.2921 | No |
| 158 | CACNA1B | calcium voltage-gated channel subunit alpha1 B [Source:HGNC Symbol;Acc:HGNC:1389] | 9092 | -0.239 | -0.2976 | No |
| 159 | MOS | "MOS proto-oncogene, serine/threonine kinase [Source:HGNC Symbol;Acc:HGNC:7199]" | 9138 | -0.243 | -0.2980 | No |
| 160 | HSPA8 | heat shock protein family A (Hsp70) member 8 [Source:HGNC Symbol;Acc:HGNC:5241] | 9140 | -0.243 | -0.2950 | No |
| 161 | RASA2 | RAS p21 protein activator 2 [Source:HGNC Symbol;Acc:HGNC:9872] | 9153 | -0.244 | -0.2928 | No |
| 162 | MRAS | muscle RAS oncogene homolog [Source:HGNC Symbol;Acc:HGNC:7227] | 9158 | -0.244 | -0.2900 | No |
| 163 | RRAS2 | RAS related 2 [Source:HGNC Symbol;Acc:HGNC:17271] | 9165 | -0.245 | -0.2873 | No |
| 164 | NF1 | neurofibromin 1 [Source:HGNC Symbol;Acc:HGNC:7765] | 9194 | -0.247 | -0.2864 | No |
| 165 | MAP3K11 | mitogen-activated protein kinase kinase kinase 11 [Source:HGNC Symbol;Acc:HGNC:6850] | 9331 | -0.258 | -0.2938 | No |
| 166 | RPS6KA5 | ribosomal protein S6 kinase A5 [Source:HGNC Symbol;Acc:HGNC:10434] | 9406 | -0.265 | -0.2963 | No |
| 167 | MAP4K3 | mitogen-activated protein kinase kinase kinase kinase 3 [Source:HGNC Symbol;Acc:HGNC:6865] | 9520 | -0.276 | -0.3016 | No |
| 168 | RELB | "RELB proto-oncogene, NF-kB subunit [Source:HGNC Symbol;Acc:HGNC:9956]" | 9550 | -0.278 | -0.3004 | No |
| 169 | DDIT3 | DNA damage inducible transcript 3 [Source:HGNC Symbol;Acc:HGNC:2726] | 9551 | -0.278 | -0.2968 | No |
| 170 | RASGRP3 | RAS guanyl releasing protein 3 [Source:HGNC Symbol;Acc:HGNC:14545] | 9603 | -0.284 | -0.2972 | No |
| 171 | FGF17 | fibroblast growth factor 17 [Source:HGNC Symbol;Acc:HGNC:3673] | 9693 | -0.291 | -0.3005 | No |
| 172 | MAPK10 | mitogen-activated protein kinase 10 [Source:HGNC Symbol;Acc:HGNC:6872] | 9699 | -0.291 | -0.2971 | No |
| 173 | FGF6 | fibroblast growth factor 6 [Source:HGNC Symbol;Acc:HGNC:3684] | 9739 | -0.296 | -0.2964 | No |
| 174 | CACNA1D | calcium voltage-gated channel subunit alpha1 D [Source:HGNC Symbol;Acc:HGNC:1391] | 9867 | -0.308 | -0.3025 | No |
| 175 | MAP2K2 | mitogen-activated protein kinase kinase 2 [Source:HGNC Symbol;Acc:HGNC:6842] | 9932 | -0.315 | -0.3035 | Yes |
| 176 | FGF4 | fibroblast growth factor 4 [Source:HGNC Symbol;Acc:HGNC:3682] | 9958 | -0.317 | -0.3014 | Yes |
| 177 | CACNB2 | calcium voltage-gated channel auxiliary subunit beta 2 [Source:HGNC Symbol;Acc:HGNC:1402] | 9967 | -0.318 | -0.2980 | Yes |
| 178 | FLNA | filamin A [Source:HGNC Symbol;Acc:HGNC:3754] | 9976 | -0.318 | -0.2945 | Yes |
| 179 | CACNB4 | calcium voltage-gated channel auxiliary subunit beta 4 [Source:HGNC Symbol;Acc:HGNC:1404] | 9992 | -0.320 | -0.2916 | Yes |
| 180 | MAP3K3 | mitogen-activated protein kinase kinase kinase 3 [Source:HGNC Symbol;Acc:HGNC:6855] | 10121 | -0.333 | -0.2974 | Yes |
| 181 | MAP4K4 | mitogen-activated protein kinase kinase kinase kinase 4 [Source:HGNC Symbol;Acc:HGNC:6866] | 10134 | -0.334 | -0.2941 | Yes |
| 182 | PLA2G2A | phospholipase A2 group IIA [Source:HGNC Symbol;Acc:HGNC:9031] | 10152 | -0.336 | -0.2911 | Yes |
| 183 | MAP2K1 | mitogen-activated protein kinase kinase 1 [Source:HGNC Symbol;Acc:HGNC:6840] | 10197 | -0.340 | -0.2902 | Yes |
| 184 | CDC25B | cell division cycle 25B [Source:HGNC Symbol;Acc:HGNC:1726] | 10206 | -0.341 | -0.2865 | Yes |
| 185 | FGF22 | fibroblast growth factor 22 [Source:HGNC Symbol;Acc:HGNC:3679] | 10261 | -0.347 | -0.2863 | Yes |
| 186 | IL1R2 | interleukin 1 receptor type 2 [Source:HGNC Symbol;Acc:HGNC:5994] | 10262 | -0.347 | -0.2818 | Yes |
| 187 | FGF12 | fibroblast growth factor 12 [Source:HGNC Symbol;Acc:HGNC:3668] | 10294 | -0.350 | -0.2798 | Yes |
| 188 | MAP3K6 | mitogen-activated protein kinase kinase kinase 6 [Source:HGNC Symbol;Acc:HGNC:6858] | 10317 | -0.353 | -0.2770 | Yes |
| 189 | FGF21 | fibroblast growth factor 21 [Source:HGNC Symbol;Acc:HGNC:3678] | 10341 | -0.355 | -0.2742 | Yes |
| 190 | MAPK8IP1 | mitogen-activated protein kinase 8 interacting protein 1 [Source:HGNC Symbol;Acc:HGNC:6882] | 10389 | -0.360 | -0.2733 | Yes |
| 191 | PRKACG | protein kinase cAMP-activated catalytic subunit gamma [Source:HGNC Symbol;Acc:HGNC:9382] | 10466 | -0.370 | -0.2746 | Yes |
| 192 | KRAS | "KRAS proto-oncogene, GTPase [Source:HGNC Symbol;Acc:HGNC:6407]" | 10544 | -0.377 | -0.2758 | Yes |
| 193 | HSPA6 | heat shock protein family A (Hsp70) member 6 [Source:HGNC Symbol;Acc:HGNC:5239] | 10561 | -0.379 | -0.2722 | Yes |
| 194 | PRKACA | protein kinase cAMP-activated catalytic subunit alpha [Source:HGNC Symbol;Acc:HGNC:9380] | 10611 | -0.384 | -0.2712 | Yes |
| 195 | PLA2G2F | phospholipase A2 group IIF [Source:HGNC Symbol;Acc:HGNC:30040] | 10628 | -0.386 | -0.2675 | Yes |
| 196 | FOS | "Fos proto-oncogene, AP-1 transcription factor subunit [Source:HGNC Symbol;Acc:HGNC:3796]" | 10640 | -0.387 | -0.2634 | Yes |
| 197 | STK3 | serine/threonine kinase 3 [Source:HGNC Symbol;Acc:HGNC:11406] | 10656 | -0.388 | -0.2596 | Yes |
| 198 | TAOK2 | TAO kinase 2 [Source:HGNC Symbol;Acc:HGNC:16835] | 10718 | -0.395 | -0.2593 | Yes |
| 199 | ELK4 | ETS transcription factor ELK4 [Source:HGNC Symbol;Acc:HGNC:3326] | 10862 | -0.411 | -0.2653 | Yes |
| 200 | MAP3K5 | mitogen-activated protein kinase kinase kinase 5 [Source:HGNC Symbol;Acc:HGNC:6857] | 10923 | -0.421 | -0.2647 | Yes |
| 201 | CHP1 | calcineurin like EF-hand protein 1 [Source:HGNC Symbol;Acc:HGNC:17433] | 10940 | -0.423 | -0.2605 | Yes |
| 202 | IKBKG | inhibitor of nuclear factor kappa B kinase regulatory subunit gamma [Source:HGNC Symbol;Acc:HGNC:5961] | 10986 | -0.428 | -0.2586 | Yes |
| 203 | RAPGEF2 | Rap guanine nucleotide exchange factor 2 [Source:HGNC Symbol;Acc:HGNC:16854] | 11041 | -0.435 | -0.2572 | Yes |
| 204 | MAPK8IP3 | mitogen-activated protein kinase 8 interacting protein 3 [Source:HGNC Symbol;Acc:HGNC:6884] | 11054 | -0.438 | -0.2526 | Yes |
| 205 | STK4 | serine/threonine kinase 4 [Source:HGNC Symbol;Acc:HGNC:11408] | 11074 | -0.441 | -0.2484 | Yes |
| 206 | PLA2G6 | phospholipase A2 group VI [Source:HGNC Symbol;Acc:HGNC:9039] | 11128 | -0.446 | -0.2469 | Yes |
| 207 | MAP3K12 | mitogen-activated protein kinase kinase kinase 12 [Source:HGNC Symbol;Acc:HGNC:6851] | 11344 | -0.478 | -0.2577 | Yes |
| 208 | NFATC4 | nuclear factor of activated T cells 4 [Source:HGNC Symbol;Acc:HGNC:7778] | 11363 | -0.481 | -0.2530 | Yes |
| 209 | PPM1B | "protein phosphatase, Mg2+/Mn2+ dependent 1B [Source:HGNC Symbol;Acc:HGNC:9276]" | 11374 | -0.482 | -0.2476 | Yes |
| 210 | MAP3K20 | mitogen-activated protein kinase kinase kinase 20 [Source:HGNC Symbol;Acc:HGNC:17797] | 11475 | -0.498 | -0.2491 | Yes |
| 211 | DUSP9 | dual specificity phosphatase 9 [Source:HGNC Symbol;Acc:HGNC:3076] | 11486 | -0.500 | -0.2434 | Yes |
| 212 | MAPK11 | mitogen-activated protein kinase 11 [Source:HGNC Symbol;Acc:HGNC:6873] | 11501 | -0.501 | -0.2381 | Yes |
| 213 | CHUK | component of inhibitor of nuclear factor kappa B kinase complex [Source:HGNC Symbol;Acc:HGNC:1974] | 11624 | -0.521 | -0.2411 | Yes |
| 214 | PRKX | protein kinase X-linked [Source:HGNC Symbol;Acc:HGNC:9441] | 11634 | -0.522 | -0.2351 | Yes |
| 215 | NR4A1 | nuclear receptor subfamily 4 group A member 1 [Source:HGNC Symbol;Acc:HGNC:7980] | 11709 | -0.535 | -0.2340 | Yes |
| 216 | DUSP2 | dual specificity phosphatase 2 [Source:HGNC Symbol;Acc:HGNC:3068] | 11768 | -0.544 | -0.2316 | Yes |
| 217 | MAPK8IP2 | mitogen-activated protein kinase 8 interacting protein 2 [Source:HGNC Symbol;Acc:HGNC:6883] | 11929 | -0.578 | -0.2369 | Yes |
| 218 | MAP3K8 | mitogen-activated protein kinase kinase kinase 8 [Source:HGNC Symbol;Acc:HGNC:6860] | 11975 | -0.589 | -0.2329 | Yes |
| 219 | ATF4 | activating transcription factor 4 [Source:HGNC Symbol;Acc:HGNC:786] | 11999 | -0.593 | -0.2271 | Yes |
| 220 | MAP2K3 | mitogen-activated protein kinase kinase 3 [Source:HGNC Symbol;Acc:HGNC:6843] | 12004 | -0.593 | -0.2198 | Yes |
| 221 | SOS1 | SOS Ras/Rac guanine nucleotide exchange factor 1 [Source:HGNC Symbol;Acc:HGNC:11187] | 12041 | -0.603 | -0.2149 | Yes |
| 222 | DUSP4 | dual specificity phosphatase 4 [Source:HGNC Symbol;Acc:HGNC:3070] | 12068 | -0.612 | -0.2091 | Yes |
| 223 | JMJD7-PLA2G4B | JMJD7-PLA2G4B readthrough [Source:HGNC Symbol;Acc:HGNC:34449] | 12076 | -0.614 | -0.2017 | Yes |
| 224 | MAPKAPK2 | MAPK activated protein kinase 2 [Source:HGNC Symbol;Acc:HGNC:6887] | 12094 | -0.619 | -0.1951 | Yes |
| 225 | PDGFA | platelet derived growth factor subunit A [Source:HGNC Symbol;Acc:HGNC:8799] | 12104 | -0.621 | -0.1879 | Yes |
| 226 | IL1B | interleukin 1 beta [Source:HGNC Symbol;Acc:HGNC:5992] | 12105 | -0.622 | -0.1799 | Yes |
| 227 | EGFR | epidermal growth factor receptor [Source:HGNC Symbol;Acc:HGNC:3236] | 12109 | -0.622 | -0.1721 | Yes |
| 228 | RASGRP2 | RAS guanyl releasing protein 2 [Source:HGNC Symbol;Acc:HGNC:9879] | 12124 | -0.625 | -0.1652 | Yes |
| 229 | MAP2K7 | mitogen-activated protein kinase kinase 7 [Source:HGNC Symbol;Acc:HGNC:6847] | 12209 | -0.649 | -0.1635 | Yes |
| 230 | MKNK2 | MAPK interacting serine/threonine kinase 2 [Source:HGNC Symbol;Acc:HGNC:7111] | 12236 | -0.655 | -0.1572 | Yes |
| 231 | HSPA1B | heat shock protein family A (Hsp70) member 1B [Source:HGNC Symbol;Acc:HGNC:5233] | 12278 | -0.670 | -0.1518 | Yes |
| 232 | DUSP8 | dual specificity phosphatase 8 [Source:HGNC Symbol;Acc:HGNC:3074] | 12303 | -0.678 | -0.1450 | Yes |
| 233 | PDGFB | platelet derived growth factor subunit B [Source:HGNC Symbol;Acc:HGNC:8800] | 12389 | -0.704 | -0.1427 | Yes |
| 234 | IL1R1 | interleukin 1 receptor type 1 [Source:HGNC Symbol;Acc:HGNC:5993] | 12419 | -0.713 | -0.1358 | Yes |
| 235 | PPP5C | protein phosphatase 5 catalytic subunit [Source:HGNC Symbol;Acc:HGNC:9322] | 12548 | -0.773 | -0.1360 | Yes |
| 236 | SRF | serum response factor [Source:HGNC Symbol;Acc:HGNC:11291] | 12549 | -0.773 | -0.1260 | Yes |
| 237 | DUSP6 | dual specificity phosphatase 6 [Source:HGNC Symbol;Acc:HGNC:3072] | 12644 | -0.821 | -0.1229 | Yes |
| 238 | PPP3R1 | "protein phosphatase 3 regulatory subunit B, alpha [Source:HGNC Symbol;Acc:HGNC:9317]" | 12653 | -0.829 | -0.1129 | Yes |
| 239 | CDC42 | cell division cycle 42 [Source:HGNC Symbol;Acc:HGNC:1736] | 12681 | -0.852 | -0.1041 | Yes |
| 240 | DUSP5 | dual specificity phosphatase 5 [Source:HGNC Symbol;Acc:HGNC:3071] | 12721 | -0.882 | -0.0959 | Yes |
| 241 | NFKB2 | nuclear factor kappa B subunit 2 [Source:HGNC Symbol;Acc:HGNC:7795] | 12750 | -0.912 | -0.0864 | Yes |
| 242 | TAOK1 | TAO kinase 1 [Source:HGNC Symbol;Acc:HGNC:29259] | 12753 | -0.918 | -0.0747 | Yes |
| 243 | MYC | "MYC proto-oncogene, bHLH transcription factor [Source:HGNC Symbol;Acc:HGNC:7553]" | 12801 | -0.970 | -0.0660 | Yes |
| 244 | JUN | "Jun proto-oncogene, AP-1 transcription factor subunit [Source:HGNC Symbol;Acc:HGNC:6204]" | 12813 | -0.994 | -0.0541 | Yes |
| 245 | GADD45B | growth arrest and DNA damage inducible beta [Source:HGNC Symbol;Acc:HGNC:4096] | 12816 | -1.001 | -0.0414 | Yes |
| 246 | JUND | "JunD proto-oncogene, AP-1 transcription factor subunit [Source:HGNC Symbol;Acc:HGNC:6206]" | 12865 | -1.116 | -0.0309 | Yes |
| 247 | DUSP1 | dual specificity phosphatase 1 [Source:HGNC Symbol;Acc:HGNC:3064] | 12884 | -1.202 | -0.0168 | Yes |
| 248 | AKT2 | AKT serine/threonine kinase 2 [Source:HGNC Symbol;Acc:HGNC:392] | 12896 | -1.404 | 0.0003 | Yes |
Table: GSEA details [plain text format]

  

Fig 2: KEGG\_MAPK\_SIGNALING\_PATHWAY      
 Blue-Pink O' Gram in the Space of the Analyzed GeneSet

  

Fig 3: KEGG\_MAPK\_SIGNALING\_PATHWAY: Random ES distribution      
 Gene set null distribution of ES for **KEGG\_MAPK\_SIGNALING\_PATHWAY**
